# *SEMA6A* overexpression inhibited tumor growth and metastasis in colorectal cancer

**DOI:** 10.1590/1414-431X2025e14896

**Published:** 2025-11-14

**Authors:** Chen Huang, Lihua Ma, Qiufang Zhao, Yongpeng Mi, Yuanyuan Tian

**Affiliations:** 1 Department of Surgery, Shijiazhuang Hospital of Traditional Chinese Medicine, Shijiazhuang, Hebei, China; 2 Department of Pulmonary Disease, Shijiazhuang Hospital of Traditional Chinese Medicine, Shijiazhuang, Hebei, China; 3 Department of Emergency Medicine, Shijiazhuang Hospital of Traditional Chinese Medicine, Shijiazhuang, Hebei, China; 4 Experimental Center, Hebei Medical University, Shijiazhuang, Hebei, China

**Keywords:** Colorectal cancer, Semaphorin 6A, Metastasis, Invasion

## Abstract

Colorectal cancer (CRC) is the fourth-leading cause of cancer-related mortality worldwide. Semaphorin 6A (SEMA6A) is a member of the semaphorin family, and its specific biological function in CRC progression remains unclear. Bioinformatics analysis revealed that *SEMA6A* expression was downregulated in CRC tissues and that low expression of *SEMA6A* was associated with a poor prognosis. Compared with those in normal colorectal epithelial cells, SEMA6A expression levels were lower in CRC cell lines. CACO2 and SW48 cells were chosen to construct stable *SEMA6A*-knockdown and *SEMA6A*-overexpressing cell lines. *SEMA6A* knockdown promoted CACO2 proliferation. Conversely, *SEMA6A* overexpression inhibited the proliferation and promoted the apoptosis of SW48 cells. Transwell and wound healing assays demonstrated that *SEMA6A* overexpression inhibited the invasion and migration ability of SW48 cells. *SEMA6A* overexpression might impede CRC cell migration and invasion by inhibiting the epithelial-to-mesenchymal transition, as evidenced by the downregulation of N-cadherin expression and the upregulation of E-cadherin expression in SW48 cells. To further validate the role of SEMA6A in CRC progression *in vivo*, transplanted tumor and liver metastasis mouse models were constructed in nude mice by injecting stable *SEMA6A*-overexpressing SW48 cell lines. *SEMA6A* overexpression inhibited tumor growth in SW48 tumor-bearing mice and the expression of Ki-67 in tumor tissues. In addition, *SEMA6A* overexpression resulted in a marked decrease in liver metastasis of CRC cells, with decreased numbers of hepatic metastatic nodules and infiltration of cancer cells. In summary, *SEMA6A* overexpression alleviated CRC progression by inhibiting tumor growth and metastasis both *in vivo* and *in vitro*.

## Introduction

Colorectal cancer (CRC) is a malignancy that affects the colon or rectum (1). CRC is predominantly asymptomatic until its advanced stages and ranks as the fourth most fatal cancer globally, resulting in over 900,000 deaths annually (2). CRC has a 25% greater incidence and mortality rate in males than in females (2). Epidemiologic studies have revealed that many factors contribute to the increased incidence of CRC, such as age, sex, inflammatory bowel disease, lifestyle, and environmental changes (3). CRC can be treated in a variety of ways, including surgical intervention, chemotherapy, and radiation therapy. These approaches can be integrated to provide a multimodal treatment strategy. However, these therapies have several adverse effects and may be cytotoxic to normal cells (4). Concurrently, these treatments may result in a diminished quality of life for the patient (2). The prognosis of CRC in patients is dismal, and these patients have a low five-year survival rate (5). To improve diagnostic and therapeutic techniques, understanding the probable molecular pathways involved in CRC and investigating the roles of critical molecules are essential.

Semaphorins are a type of transmembrane protein (6). The semaphorin family includes more than 20 members. The members are divided into eight classes on the basis of their structure and amino acid sequence, and all eight classes contain a conserved Sema-PSI structural domain (6). Several semaphorins have been demonstrated to modulate tumor angiogenesis, cancer cell invasion, and metastasis (7). The class 6 transmembrane semaphorin proteins have the longest cytoplasmic domain, with approximately 400 amino acids (6). Semaphorin 6A (SEMA6A) belongs to class 6 of the semaphorin family, and its functions in axon guidance (8), angiogenesis (9), and neuronal migration (10) have been clearly revealed. The absence of SEMA6A reduces myelination in cocultures of neurons/oligodendrocytes (11). *SEMA6A* overexpression in the cerebral cortex promotes recovery after cerebral ischemia in rats (12). The role of SEMA6A in the development and progression of cancer has also been reported in recent years (13,14). *SEMA6A* expression was downregulated in lung cancer tissues, and its overexpression inhibited lung cancer cell proliferation both *in vitro* and *in vivo* (15). Certain semaphorin proteins have been shown to exhibit antitumor or protumor effects in different tumors (16). Consequently, we posited that SEMA6A might play distinct roles in different cancers.

Currently, the role of SEMA6A in CRC has yet to be elucidated. A bioinformatics study reported that *SEMA6A* expression is significantly downregulated in colon cancer (17). These findings prompted us to investigate the role of SEMA6A in CRC.

## Material and Methods

### Bioinformatics

Four datasets related to CRC were acquired from the NCBI Gene Expression Omnibus (GEO) database (https://www.ncbi.nlm.nih.gov/geo/): GSE39582, GSE23878, GSE8671, and GSE25071 (Table 1). The TNMplot database (https://tnmplot.com/analysis/) was used to investigate the expression of the *SEMA6A* gene in colorectal tumor tissues and normal colorectal tissues. We explored overall survival (OS) and postprogression survival (PPS) associated with the *SEMA6A* gene in CRC patients utilizing the Kaplan-Meier plotter (https://kmplot.com/analysis/index.php?p=background) survival analysis tool.

**Table 1:** Information about the 4 datasets on colorectal cancer (CRC).

Datasets	CRC tumor tissue (n)	Normal tissue (n)
GSE39582	566	19
GSE23878	35	24
GSE8671	32	32
GSE25071	46	4

### Cell culture and transfection

The human normal colorectal epithelial cell line (NCM460) and CRC cell lines (HCT116, SW620, CACO2, SW48, and LoVo) were purchased from Cellverse (China) and cultured in RPMI-1640 medium, McCoy's 5A medium, L15 medium, MEM, DMEM, and F12K medium containing 10% fetal bovine serum, respectively. To investigate the function of SEMA6A in CRC, the CACO2 cell lines were transfected with short hairpin RNAs targeting *SEMA6A* (*SEMA6A* ^sh-1^ and *SEMA6A* ^sh-2^) or a negative control (NC^sh^). The SW48 cell line was transfected with the *SEMA6A* overexpression (*SEMA6A* ^oe^) plasmid or empty vector. The cells were treated with G418 solution (200 mg/mL) for screening stable expression cell lines. Subsequent *in vivo* and *in vitro* experiments were performed with stable expression cell lines.

### Animal model establishment

Female BALB/c nude mice were purchased from Huachuang Sino (China) and housed in a specific pathogen-free (SPF) environment. To construct the transplanted tumor model of SW48, a SW48 cell suspension (1.5×10^6^/50 μL) (18) stably expressing the empty vector or *SEMA6A* ^oe^ was injected subcutaneously into the right axilla of each mouse. The length and width of the mouse tumors were measured every 5 days, and the volume was calculated by the following formula: length × width^2^ / 2. The mice were sacrificed on day 35, and the subcutaneous tumors were isolated, photographed, and weighed. The construction of the CRC liver metastasis model was performed as previously reported (19). Nude mice were anesthetized and their abdomens were sterilized. A small incision was made on the left side of the abdomen of each nude mouse. The spleen was gently removed with tissue forceps, and SW48 cells (2×10^6^/50 μL) (20) stably expressing the vector or *SEMA6A* ^oe^ were slowly injected into the spleen. After the injection, the spleen was returned to its original position, and the wound was sutured layer by layer. The livers of the mice were isolated and photographed after 10 weeks. The number of metastatic nodules in the liver was recorded. All the animal experiments were approved by the Ethics Committee of Shijiazhuang Hospital of Traditional Chinese Medicine.

### Western blot

Total protein was extracted from the cells, and the protein concentration was quantified with a BCA kit (Solarbio, China). Proteins were separated by sodium dodecyl sulfate-polyacrylamide gel electrophoresis (SDS-PAGE) and transferred to polyvinylidene fluoride (PVDF) membranes. The protein-free sites on the PVDF membranes were blocked with a blocking solution (Solarbio). The PVDF membranes were subsequently incubated with a SEMA6A antibody (1:1000, Invitrogen, USA) or a GAPDH antibody (1:10000, Proteintech, China) overnight at 4°C. The PVDF membranes were then incubated with goat anti-rabbit IgG-HRP (1:3000, Solarbio) or goat anti-mouse IgG-HRP (1:3000, Solarbio) for 1 h at 37°C. Finally, the protein bands were visualized with an ECL solution (Solarbio).

### Quantitative real-time PCR (qPCR)

Total RNA from CACO2 and SW48 cells was extracted by TRIZOL (BioTeke, China) and subsequently reverse transcribed into complementary DNA using All-in-One First-Strand SuperMix (Magen Biotechnology, China). qPCR was performed using Pangaea 3 (Aperbio, China). GAPDH was used as an internal reference, and the relative expression levels of mRNAs were evaluated using the 2^-ΔΔCT^ method. The primers used in the present study were as follows: *SEMA6A*, forward: CGGGCGTGATGTTGTCC; reverse: AGCGGGTGCGTCTTGAT; and GAPDH, forward: GACCTGACCTGCCGTCTAG; reverse: AGGAGTGGGTGTCGCTGT.

### CCK-8 assay

CACO2 and SW48 cells were seeded in 96-well plates at 5×10^3^ cells per well. The cells were incubated in an incubator for 0, 24, 48, or 72 h, and then, CCK-8 (10 μL/well, Biosharp Life Sciences, China) was added to the wells and incubated for 2 h. The absorbance values of each well at 450 nm were measured, and the data were analyzed to evaluate the proliferation ability of the cells.

### Flow cytometry

The cell proliferation of CACO2 and SW48 cells was detected with EdU staining (Elabscience, China). EdU staining solution (10 μM) was coincubated with CACO2 and SW48 cells. The cells were collected, washed with 1% BSA, and then fixed with 4% paraformaldehyde (PFA) for 15 min at room temperature (RT, 25-27°C). The cells were permeabilized by incubation with 0.5% Triton X-100 for 20 min at RT. The cells were treated with Click-iT reaction mixture for 30 min at RT and subsequently analyzed using NovoCyte (Agilent, USA).

Apoptosis in SW48 cells was assessed by an apoptosis detection kit (Biosharp Life Sciences). The cells were incubated with Annexin V-FITC for 10 min at RT and then incubated with propidium iodide (PI) for 5 min at RT, followed by flow cytometry.

### Transwell assay

Matrigel (USA) was diluted with serum-free medium and spread evenly in the upper chambers of Transwell cell culture chambers (LABSELECT, China). Transwell chambers were placed into 24-well plates and used after the solidification of the Matrigel. The cells were prepared as single-cell suspensions with serum-free medium. A cell suspension (200 μL) was added to the upper chamber of the Transwell system, and medium containing 10% FBS (800 μL) was added to the lower chamber. The cells were cultured for 24 h, fixed with 4% PFA (Aladdin, China) for 20 min at RT and stained with crystal violet staining solution (Amresco, USA) for 2 min. The migration and invasion of the cells were observed under a light microscope.

### Wound healing assay

CACO2 and SW48 cells were treated with mitomycin C (MCE, USA) prior to the experiments to inhibit cell proliferation. The cell layer surface was scratched with a 200-µL pipette tip. The cells were cultured in serum-free medium. Cell migration was observed under a light microscope at 0 and 24 h.

### Immunofluorescence staining

CACO2 and SW48 cells were fixed with 4% PFA and permeabilized with 0.1% Triton X-100. The cells were subsequently blocked with 1% BSA for 15 min at RT. The cells were incubated with primary antibodies against N-cadherin (1:200, Proteintech) or E-cadherin (1:200, Proteintech) overnight at 4°C. The secondary antibody Cy3-goat Anti-rabbit IgG (1:200, Proteintech) was incubated with the cells for 1 h at RT. DAPI was used to stain the nuclei. Finally, the cells were observed and photographed under a microscope.

### Immunohistochemical (IHC) and hematoxylin-eosin (H&E) staining

IHC was performed to detect Ki-67 expression in SW48 tumor tissues. The tumor tissue was embedded in paraffin and cut into 5-μm slices. The tissue sections were deparaffinized to water, and the tissue was repaired with antigen repair solution. The sections were incubated with 3% H_2_O_2_ for 15 min at RT to eliminate endogenous peroxidase activity. The sections were blocked with 1% BSA at RT for 15 min. The tissue sections were incubated with the primary antibody Ki-67 (1:50, Affinity, China) overnight at 4°C and then with secondary antibody goat anti-rabbit IgG (1:100, Solarbio) for 45 min at RT. The immunocomplexes were visualized with diaminobenzidine (DAB, Sangon, China) as the chromogen. The tissues were restained with hematoxylin (Solarbio). Finally, the tumor slices were observed under a microscope.

H&E staining was performed to detect hepatic metastasis in SW48 tumor-bearing mice. The SW48 tumor tissues were embedded in paraffin and cut into 5-μm slices. The tissue sections were deparaffinized in water and then stained with H&E (Sangon). The slices were observed under a microscope.

### Statistical analyses

Measurement data are reported as means±SD. Comparisons between two groups were performed using unpaired *t*-tests or Mann-Whitney tests, and comparisons among multiple groups were performed using one-way analysis of variance (ANOVA) or two-way ANOVA. A difference in the data with a value of P<0.05 was considered statistically significant.

## Results

### *SEMA6A* expression was decreased in CRC datasets

**Figure 1:**
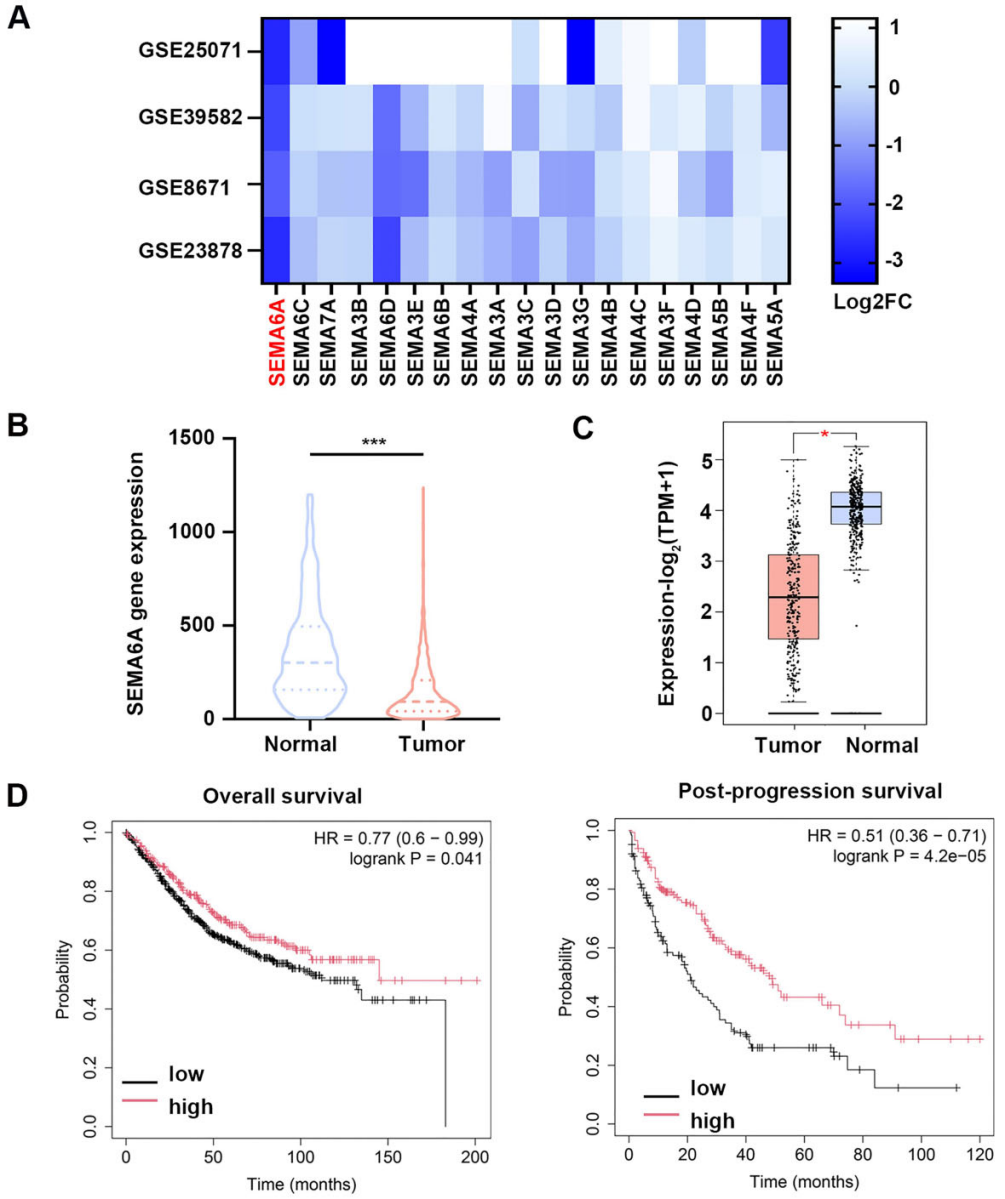
Semaphorin 6A (*SEMA6A*) expression was decreased in colorectal cancer (CRC) databases. **A**, Heatmap of semaphorin family expression in GSE25071, GSE23878, GSE8671, and GSE39582. **B**, Violin plot of *SEMA6A* gene expression in normal colorectal tissues and CRC tissues. Data were obtained from TNMplot.com. **C**, The differential mRNA expression of *SEMA6A* between normal colorectal tissues and CRC tissues was based on the GEPIA database. **D**, The left panel shows the Kaplan-Meier curve of overall survival (OS) of *SEMA6A* expression in CRC. The right panel shows the Kaplan-Meier curve of post-progression survival (PPS) of *SEMA6A* expression in CRC. Data are reported as median and interquartile range. *P<0.05, ***P<0.001 (*t*-test or Mann-Whitney test).

The semaphorin family has been shown to play a significant role in the development and progression of cancer, but findings concerning the semaphorin family in CRC are limited. We analyzed the expression of the semaphorin family in four CRC-related datasets. The *SEMA6A*, *SEMA7A*, *SEMA3G*, and *SEMA5A* genes were markedly downregulated in the datasets, and the *SEMA6A* gene exhibited the most significant downregulation (Figure 1A). To further determine the expression of SEMA6A in CRC, we analyzed SEMA6A using the TNMplot database. *SEMA6A* expression was significantly lower in CRC samples than in normal samples (Figure 1B). This result was further supported by analysis using the GEPIA2 database (Figure 1C). Kaplan-Meier survival curve analysis was performed to assess the relationship between *SEMA6A* expression and the prognosis of CRC in patients (Figure 1D). CRC patients with low *SEMA6A* expression presented inferior OS and PPS than did those with high *SEMA6A* expression (Figure 1D). Given the substantial differential expression of *SEMA6A* in CRC-related datasets and its robust association with CRC prognosis, SEMA6A might be pivotal in the progression of CRC. Consequently, we further investigated the role of SEMA6A in CRC.

### Effects of SEMA6A on CRC cell proliferation and apoptosis

**Figure 2:**
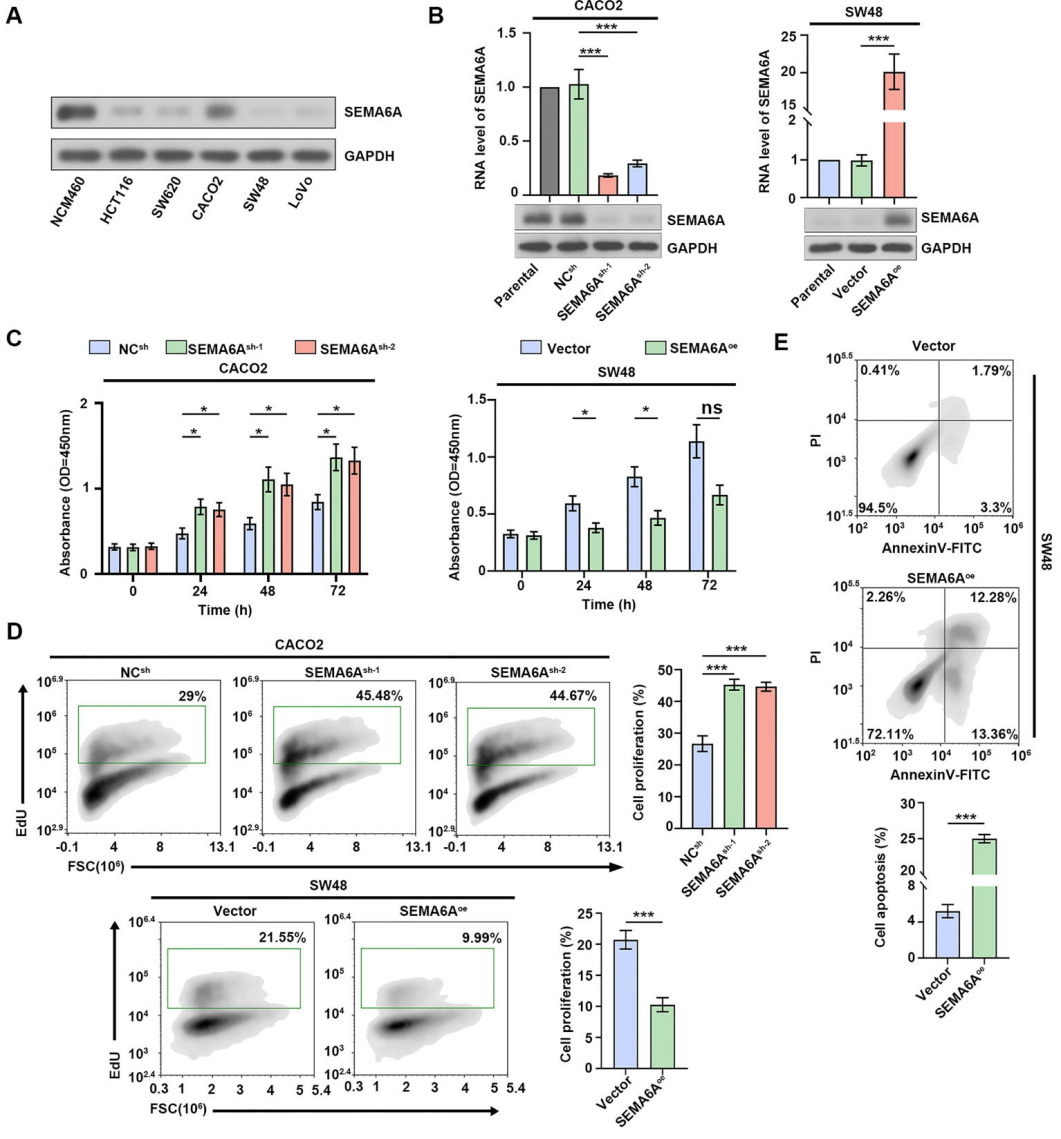
Effects of SEMA6A on colorectal cancer (CRC) cell proliferation and apoptosis. **A**, Immune blots of SEMA6A in normal colorectal cells and different CRC cells. **B**, RNA levels and immune blots of SEMA6A in CACO2 and SW48. **C**, CCK8 assay of CACO2 and SW48 at 0, 24, 48, and 72 h. **D**, Detection of cell proliferation in CACO2 and SW48 by flow cytometry. **E**, Detection of apoptosis in SW48 by flow cytometry. Data are reported as means and SD. *P<0.05, ***P<0.001 (ANOVA). ns: no significance.

SEMA6A expression was lower in the five CRC cell lines than in the normal cell line. Compared with other CRC cells, SEMA6A had the highest protein expression in the CACO2 cells and the lowest protein expression in the SW48 cells (Figure 2A). Stably transfected CRC cell lines with *SEMA6A* knockdown or overexpression were successfully constructed from CACO2 cells or SW48 cells (Figure 2B). First, the effects of *SEMA6A* on the proliferation and apoptosis of CRC cells were investigated. *SEMA6A* knockdown markedly enhanced CACO2 cell proliferation, whereas *SEMA6A* overexpression inhibited SW48 cell proliferation (Figure 2C). Flow cytometry of EdU staining further corroborated these findings (Figure 2D). Moreover, *SEMA6A* overexpression promoted the apoptosis of SW48 cells (Figure 2E). Taken together, these results suggested that *SEMA6A* overexpression inhibited proliferation and promoted apoptosis in CRC cells.

### *SEMA6A* overexpression inhibited CRC cell migration and invasion *in vitro*

**Figure 3:**
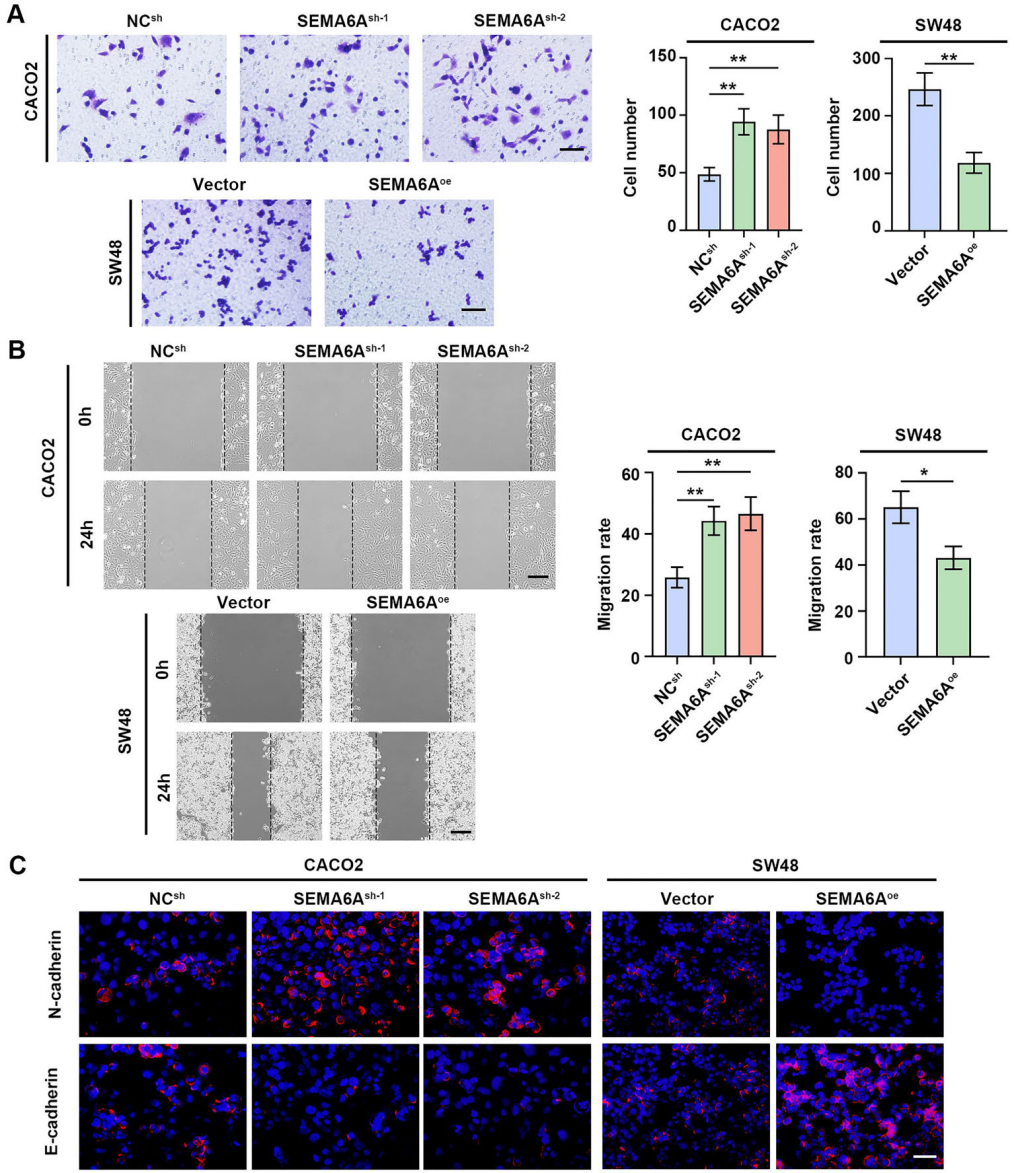
*SEMA6A* overexpression inhibited CRC cell migration and invasion *in vitro*. **A**, Effects of SEMA6A on the invasion ability of CACO2 and SW48 detected by the transwell assay (scale bar=100 μm). **B**, The effect of SEMA6A on the migration of CACO2 and SW48 was detected by wound healing assay (scale bar=200 μm). **C**, The expression of N-cadherin and E-cadherin in CACO2 and SW48 was detected by immunofluorescence staining (scale bar=50 μm). Data are reported as means and SD. *P<0.05, **P<0.01 (ANOVA).

The migration and invasion of tumor cells promote cancer progression (21). Transwell assays revealed that knockdown of *SEMA6A* enhanced the migration and invasion of CACO2 cells, whereas *SEMA6A* overexpression inhibited migration and invasion of SW48 cells (Figure 3A). Wound healing was significantly inhibited by *SEMA6A* overexpression in SW48 cells, whereas *SEMA6A* knockdown markedly enhanced the migration of CACO2 cells (Figure 3B). The epithelial-to-mesenchymal transition (EMT) is considered a key mechanism involved in cancer progression and metastasis (22). An IF assay was used to detect EMT-related indicators. *SEMA6A* knockdown increased N-cadherin-positive cell signals in CACO2 cells, whereas E-cadherin-positive signals were markedly diminished (Figure 3C). However, *SEMA6A* overexpression inhibited the activation of the EMT in SW48 cells (Figure 3C). These results suggested that the overexpression of *SEMA6A* suppressed the migration and invasion of CRC cells *in vitro*.

### *SEMA6A* overexpression suppressed CRC tumor growth in mice

**Figure 4:**
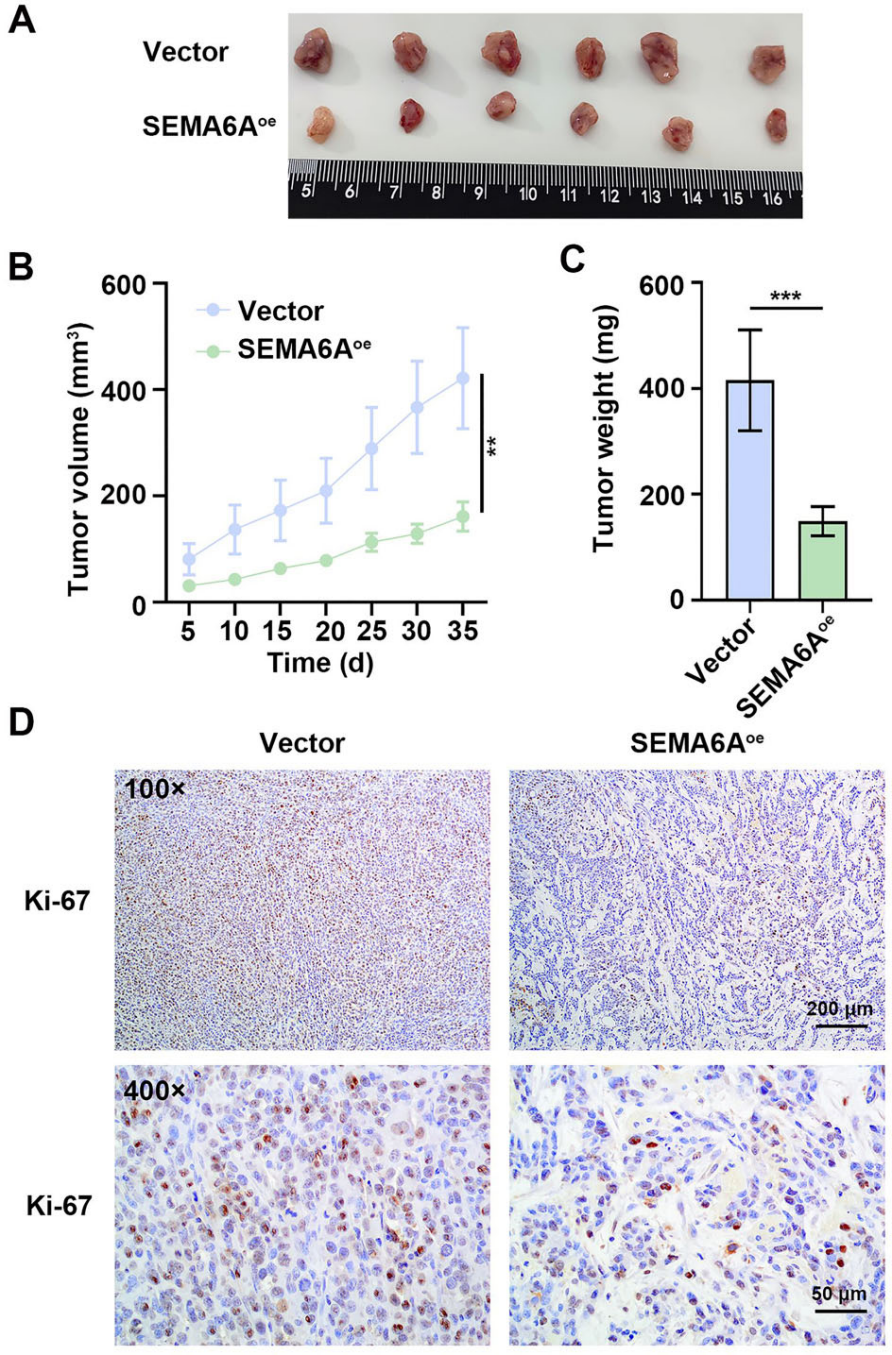
*SEMA6A* overexpression suppressed colorectal cancer (CRC) tumor growth in mice. **A**, Images of SW48 tumors separated from euthanized mice. **B**, The SW48 tumor volume was measured at different time points. **C**, Weight of the SW48 tumors. **D**, Representative images of immunohistochemical detection of Ki-67 expression in tumor tissues (scale bar=200 or 50 μm). Data are reported as means and SD. **P<0.01, ***P<0.001 (*t*-test).

We subsequently explored the role of SEMA6A in CRC tumor growth *in vivo*. As shown in Figure 4A-C, the tumor volume and weight of SW48 tumor-bearing mice were significantly inhibited by *SEMA6A* overexpression. Ki-67 is a proliferation marker for malignant tumors (23). Ki-67 expression was reduced in the tumor tissues of the mice with *SEMA6A* overexpression (Figure 4D). The overexpression of *SEMA6A* significantly inhibited the growth of CRC tumors *in vivo*.

### *SEMA6A* overexpression inhibited liver metastasis of CRC in mice

**Figure 5:**
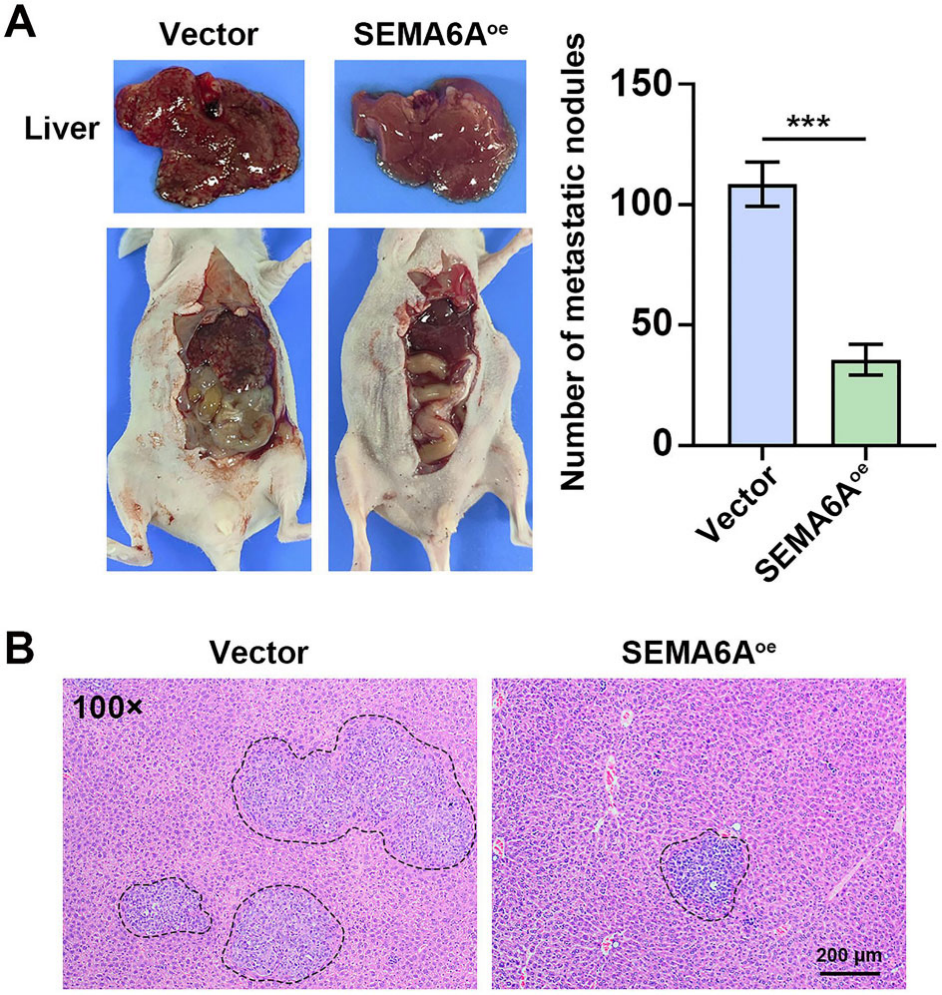
*SEMA6A* overexpression inhibited liver metastasis of colorectal cancer (CRC) in mice. **A**, Left panel: Representative images of SW48 tumor-bearing mice with liver metastasis. Right panel: number of metastatic nodules in SW48 tumor-bearing mice. **B**, Representative images of H&E staining of liver tissue collected from SW48 tumor-bearing mice. Cancer cell infiltration is outlined by the black dotted line (scale bar=200 μm). Data are reported as means and SD. ***P<0.001 (*t*-test).

Translucent scattered metastatic nodules were observed in the livers of mice with CRC metastasis, and the number of liver nodules was significantly increased (Figure 5A). This phenomenon was inhibited by *SEMA6A* overexpression. The results of H&E staining further supported the above findings, and *SEMA6A* overexpression reduced the infiltration of tumor cells into the livers of the mice (Figure 5B). These results suggested that *SEMA6A* overexpression inhibited the liver metastasis of CRC tumors.

## Discussion

The morbidity and mortality rates of CRC remain high, imposing a significant health burden globally. Although developments in modern medicine have prolonged the survival of CRC patients to some extent, the prognosis of CRC in patients remains poor. Consequently, exploring the functions of key molecules in CRC is essential.

In clear cell renal cell carcinoma (ccRCC), *SEMA6A* expression is upregulated, and its overexpression promotes the malignant phenotype of ccRCC (13). Moreover, *SEMA6A* overexpression prompted proliferation and markedly enhanced invasiveness in melanoma cells (14). However, the increase in *SEMA6A* expression impedes the development and progression of lung cancer (24). On the basis of the above reports, the function of *SEMA6A* might be complex as it plays different roles in different tumors. To elucidate the possible role of *SEMA6A* in CRC, bioinformatics was utilized. Bioinformatics analysis indicated that *SEMA6A* expression was markedly downregulated in patients with CRC. CRC patients with higher *SEMA6A* expression have more favorable outcomes than those with lower *SEMA6A* expression. Moreover, decreased expression of *SEMA6A* in multiple CRC cell lines was observed. As a result, we hypothesized that *SEMA6A* might be involved in the development of CRC as an inhibitory factor. Relevant experiments further confirmed that the overexpression of *SEMA6A* inhibited CRC cell proliferation and metastasis both *in vitro* and *in vivo*.

Members of the class 6 semaphorin family include SEMA6A, SEMA6B, SEMA6C, and SEMA6D. According to previous reports, the 6 semaphorins might exert different effects on cell proliferation or apoptosis by transmitting distinct signals in different cells. *SEMA6A* activated pro-survival and pro-proliferative cell signaling in melanoma cells (14). *SEMA6A* regulates the remodeling of the actin cytoskeleton, thereby maintaining the invasive behavior of melanoma cells (25). A reduction in *SEMA6A* mRNA expression inhibited the progression of hepatocellular carcinoma (26). Downregulation of *SEMA6A* induced apoptosis in human oral cancer cells (27). In these studies, *SEMA6A* was identified as a factor that promotes cancer progression. Nevertheless, Shen et al. (15) reported that *SEMA6A* expression was downregulated in lung cancer tissues and that *SEMA6A* overexpression inhibited lung cancer cell proliferation both *in vitro* and *in vivo*. *SEMA6A* inhibited the migration ability of lung cancer cells through the NRF2/HMOX1 axis, and *SEMA6A* overexpression significantly reduced the migration of lung cancer cells (28). *SEMA6B* overexpression in hepatocytes inhibited proliferation and induced apoptosis through Go/G1 cell cycle block (29). However, *SEMA6B* can induce pro-proliferative signaling to promote cell proliferation in U87MG glioblastoma cells (30). These reports suggest that the role of the 6-class semaphorin family in cancer is distinct depending on the type of cancer and the specific mechanism involved. In our study, *SEMA6A* overexpression inhibited CRC cell proliferation and promoted the apoptosis of CRC cell lines *in vivo* and *in vitro*. Our study provided evidence that SEMA6A inhibited cell proliferation and promoted apoptosis in CRC cells.

CRC has a significant incidence of recurrence and metastasis, which are the primary contributors to patient death. An enhanced understanding of the molecular pathways that regulate the local invasion and distant metastasis of CRC may facilitate the development of treatment strategies for patients with CRC. SEMA6A was initially characterized as a modulator of granulocyte and endothelial cell migration (31,32). Silencing SEMA6A facilitates lung cancer cell migration (28). In the present study, we demonstrated that the overexpression of *SEMA6A* inhibited the migration of CRC cells. The transformation of epithelial cells into motile mesenchymal cells is known as the EMT (33). EMT is believed to be a prerequisite step for initial tumor cells to become active and invasive, which leads to the metastasis and recurrence of many cancers (34). Most human CRC cell lines exhibit a partial EMT, which is a state that promotes CRC metastasis (35). The occurrence of the EMT greatly increases the difficulty of treating metastatic CRC. SEMA6D regulates the proliferation, migration and invasion of breast cancer cell lines through the EMT (36). These findings suggested that SEMA6A could also potentially play a role in the EMT. Therefore, we detected EMT-related indicators, and the results suggested that *SEMA6A* overexpression *in vitro* might attenuate the invasion and migration ability of CRC cells by inhibiting the EMT. The liver is the most common target of hematogenous CRC metastasis and the most prominent site leading to patient death from this malignancy (37). We established a model of CRC liver metastasis and demonstrated that the overexpression of *SEMA6A* also inhibited CRC metastasis *in vivo*.

Previous studies have generally focused on the pro-cancer effects of SEMA6A in various cancers, including malignant glioma and hepatocellular carcinoma. However, this study demonstrates for the first time that SEMA6A has anticancer effects on CRC through both *in vivo* and *in vitro* experiments. The present study also has limitations. Although bioinformatics analysis suggested a correlation between SEMA6A expression and CRC prognosis, we lacked clinical samples for validation to fully establish this correlation. This study revealed that SEMA6A inhibits CRC progression. However, the underlying molecular mechanisms, such as direct interactions or downstream factors, remain to be elucidated. Further investigations of these mechanisms are planned for the future.

In the majority of cancer types, elevated levels of SEMA6A are associated with poorer overall prognosis and increased cancer cell invasiveness. This phenomenon has been confirmed in malignant glioma (38), hepatocellular carcinoma (39), renal cell carcinoma (40), and melanoma (14). However, our research provides new evidence that *SEMA6A* overexpression alleviates the malignant progression of CRC. *SEMA6A* overexpression inhibited CRC cell proliferation and might alleviate the migration and invasion of CRC cells *in vitro* by suppressing the EMT. Similarly, *SEMA6A* overexpression inhibited the growth and metastasis of CRC tumors *in vivo*. Our study suggested that SEMA6A might be a potential therapeutic target for CRC.

## Data Availability

All data generated or analyzed during this study are included in this published article.
